# Association of hepatitis B virus genomes with active chromatin hubs challenges host replication fidelity, leading to DNA damage

**DOI:** 10.1128/jvi.01014-25

**Published:** 2025-10-10

**Authors:** Gavin J. Marcoe, Clairine I. S. Larsen, Monnette F. Summers, Kinjal Majumder

**Affiliations:** 1 Cancer Biology Graduate Program, Madison, Wisconsin, USA; 2 Institute for Molecular Virology 70033, Madison, Wisconsin, USA; 3 Cellular and Molecular Biology Graduate Program, Madison, Wisconsin, USA; 4 McArdle Laboratory for Cancer Research 219455 , Madison, Wisconsin, USA; 5 University of Wisconsin School of Medicine and Public Health 5232, Madison, Wisconsin, USA; 6 University of Wisconsin Carbone Cancer Center 206022, Madison, Wisconsin, USA; Wake Forest University School of Medicine, Winston-Salem, North Carolina, USA

**Keywords:** hepatitis B virus, DNA damage response, replication stress, chromosome conformation capture

## Abstract

Hepatitis B virus (HBV), the leading cause of liver cancer, infects almost 300 million individuals worldwide. Although HBV-infected patients benefit from drug regimens that help control chronic infection, they are rarely clinically cured of HBV. The HBV genome persists in the nucleus of infected hepatocytes as a covalently closed circular DNA (cccDNA) molecule, a reservoir of HBV DNA molecules that serves as the template for reactivation of long-term chronic HBV. Despite playing a central role in the viral life cycle, little is understood about where cccDNA molecules localize, why they are so stable, and how they impact the host nuclear compartment. Here, we show that HBV genomes in cell line models provoke cellular replication stress early in infection, which culminates in global DNA damage response (DDR) signals. HBV-induced replication stress early in infection correlates with the onset of virus replication and expression of viral genes, forming viral genome reservoirs in distinct subnuclear compartments. Using a novel high-throughput chromosome conformation capture technology that monitors the localization of HBV cccDNA molecules in few cells, we show that cccDNA molecules persist in the vicinity of transcriptionally active cellular promoters. Most of these sites contain binding elements for the stress-response protein DDIT3 (DNA damage inducible transcript 3). RNAi-mediated knockdown of DDIT3 rescues HBV-induced replication stress without altering the cccDNA reservoir. Our findings contribute to the understanding of how HBV’s navigation of the host nuclear environment regulates genome stability, identifying functional targets for development of therapies against HBV-induced liver cancer.

**IMPORTANCE:**

Hepatitis B virus (HBV) is the leading infectious cause of liver cancer globally. The virus persists in the nucleus long term by forming reservoirs in human liver cells. We have discovered that HBV DNA localizes to sites on the host genome associated with transcriptionally active chromatin, and in doing so, HBV interferes with the host’s ability to efficiently undergo amplification. This results in the induction of cellular DNA breaks, which we propose contributes to eventual cancer progression. Our findings provide new insights into how HBV infection may lead to liver cancer.

## INTRODUCTION

Nearly 300 million people across the globe suffer from chronic hepatitis B virus (HBV) infection. Every year, over 800,000 deaths can be attributed to the effects of chronic infection, such as hepatocellular carcinoma (liver cancer) or liver cirrhosis. A significant number of new infections are caused by vertical transmission, from the mother to child during pregnancy or upon breastfeeding (1). Once infected, hepatitis B virions enter hepatocytes via receptor-mediated endocytosis using the sodium taurocholate co-transporting polypeptide (NTCP) entry receptor. Once inside the cytoplasm, the viral genome in the form of relaxed circular DNA (rcDNA) undergoes nuclear import to enter the host cell’s nucleus (2). In the nucleus, rcDNA is converted into the covalently closed circular DNA (cccDNA) with the aid of host DNA repair proteins (3, 4). This form of the viral genome plays a central role in the life cycle of the virus, where cccDNA acts as a transcription template to produce several RNA forms including precore RNA, which is translated into the hepatitis B E-antigen [HBeAg; (5, 6)]. Unfortunately, little is known about where the HBV genome localizes, how it utilizes host factors, and how it impacts the stability of the host genome. Understanding these critical aspects of HBV’s navigation of the nuclear environment will help identify functional targets to fully eliminate HBV infection from the liver. While there is an HBV vaccine, antiviral therapies are not curative due to persistence of cccDNA in hepatocytes and lack of widely available drugs that target cccDNA molecules (7–9). Therefore, it is critical to understand where molecules of the HBV genome persist in the nuclear milieu long term and how HBV genomes alter the host environment.

Viruses provoke a cellular DNA damage response (DDR) in the host nuclear compartment, generated by viral genomes, transcripts, and proteins that are expressed during infection. This cellular DDR is amplified by the phosphatidylinositol 3-kinase-related kinases ATM, ATR, and DNA-PK, which can have proviral or antiviral effects (10). As a result, viruses have evolved distinct strategies to usurp or inactivate host DDR signals. Tumor viruses like human papillomavirus (HPV), polyomaviruses, and Epstein-Barr virus (EBV) are regulated by ATM/ATR signaling (11–18), whereas other DNA viruses like adenoviruses and herpes simplex virus (HSV) have a more complicated relationship with the host DDR signaling kinases (19–22). The single-stranded DNA parvovirus adeno-associated virus (AAV) induces a DNA-PK-dependent cellular DDR signaling cascade (23), whereas its relative minute virus of mice (MVM) induces an ATM-dependent DDR (24). Interestingly, the life cycle of HBV in host hepatocytes is dependent on pan-nuclear ATM and ATR signaling (25, 26). Additionally, the HBV core protein HBc is a substrate for ATM phosphorylation, implicating ATM-mediated signaling events in regulating the HBV life cycle (27). Although cellular ATM and ATR signaling activate chromatin modifiers, downstream kinases, host polymerases, and DNA repair pathways in the host nuclear environment (10), it remains unknown which aspect of ATM/ATR signaling regulates the HBV life cycle in the nuclear compartment.

HBV genomes enter the host nuclear environment in the form of partially single- and partially double-stranded DNA molecules called relaxed circular DNA (rcDNA). Conversion of rcDNA into cccDNA is carried out by host replication and repair proteins such as DNA polymerase kappa, Flap endonuclease 1 (FEN1), and host DNA ligases (8, 28–30). Upon formation, cccDNA molecules act as transcription templates, generating pregenomic RNA (pgRNA), messenger RNAs (mRNAs), and precore RNA. The pgRNA undergoes reverse transcription within newly created nucleocapsids to form more rcDNA molecules that generate progeny infectious particles in the cytoplasm (6). Expression of HBV genes produces HBx, HBc, E antigen (HBeAg), polymerase, and the small (S), medium (M), and large (L) surface proteins in infected cells (31). Out of these viral factors, only the viral polymerase is known to associate with the viral origins to facilitate replication. Additionally, the HBV oncoprotein HBx interacts with host structural maintenance of chromosomes (SMC) proteins SMC5/6 that are required for efficient cellular DDR signals leading to homologous recombination (HR) (32). Since HR signaling in the cell is regulated by ATM and ATR kinases, these findings suggest that HBV likely utilizes cellular DDR pathways through HBx. Consistent with this assertion, ATR-mediated signals have also been implicated in facilitating cccDNA formation (33). However, it remains unknown how these local ATR signals are activated by HBV and what the consequences are on the host genome.

One of the effects of virus-induced DDR is the regulation of cell cycle entry or induction of cell cycle arrest. DNA tumor viruses such as HPV (34), Kaposi’s sarcoma-associated herpesvirus (KSHV) (35, 36), EBV (37), and human T-lymphotropic virus 1 (HTLV1) (38) antagonize cell cycle checkpoints to cause neoplastic transformation in infected cells. Paradoxically, while infection of primary human hepatocytes with HBV leads to G2/M arrest (39), infection of transformed HepG2 cells with HBV causes G1/S arrest (40). A likely unifying mechanism for HBV-induced cell cycle dysregulation is through the HBx protein that dysregulates cell cycle checkpoint controls (41). However, the timing of when HBV-induced genome instability is initiated and the signaling events that drive cell cycle dysregulation remain unknown.

The cellular genome contains regions that preferentially accrue DNA damage called fragile sites, generated by replication stress (termed early replicating fragile sites [42, 43]) or formation of secondary structures in late-replicating DNA regions (termed common fragile sites [44–46]). Both types of cellular fragile genomic regions are caused by or lead to transcription-replication conflicts, partially because these regions are also correlated with transcriptionally active chromatin. Cellular fragile sites are sites of localization of diverse DNA viruses, including HPV (47, 48), MVM (49), and EBV (50). Interestingly, HPV genomes are tethered to genomic fragile sites using host chromatin factors such as bromodomain-containing protein 4 (BRD4), whereas EBV and MVM induce DNA damage at fragile genomic regions through unknown mechanisms (47, 51, 52). Prior studies investigating the localization of HBV genomes to cellular sites have discovered that transcriptionally inactive forms of HBV localize to distinct active chromatin hubs that package the human genome (53). Independently, HBV localization sites on the human genomes are enriched in binding sites of the cellular transcription factor Yin-Yang 1 (YY1) (7, 54). However, it remains unclear what form of the viral genome associates with these cellular sites. Additionally, the cause-effect relationship between host chromatin, genome stability, and HBV genome localization remains unknown.

In this study, we investigated the mechanisms by which HBV perturbs host genome stability and associates with the cellular chromatin. We show that HBV-induced replication stress is initiated early after infection and exacerbates over time. As infection progresses, this replication stress leads to the induction of cellular DDR signals that are in spatial proximity to viral genomes. HBV genomes, including the cccDNA reservoir, associate with a subset of transcriptionally active promoter regions, many of which are enriched in binding sites of cellular transcription factors, such as the DNA damage inducible transcript 3 (DDIT3), a stress response marker that is known to be associated with liver cancer (55). Absence of DDIT3 in virus-infected cells leads to the rescue of HBV-induced replication stress, suggesting DDIT3 molecules at HBV-associated cellular sites play a role in regulating genome stability. Our findings illuminate the complex interplay between host cell chromatin, DDR machinery, and viral genomes that regulate HBV-induced DNA damage.

## RESULTS

### HBV infection provokes DDR signals by Day 5

**Fig 1:**
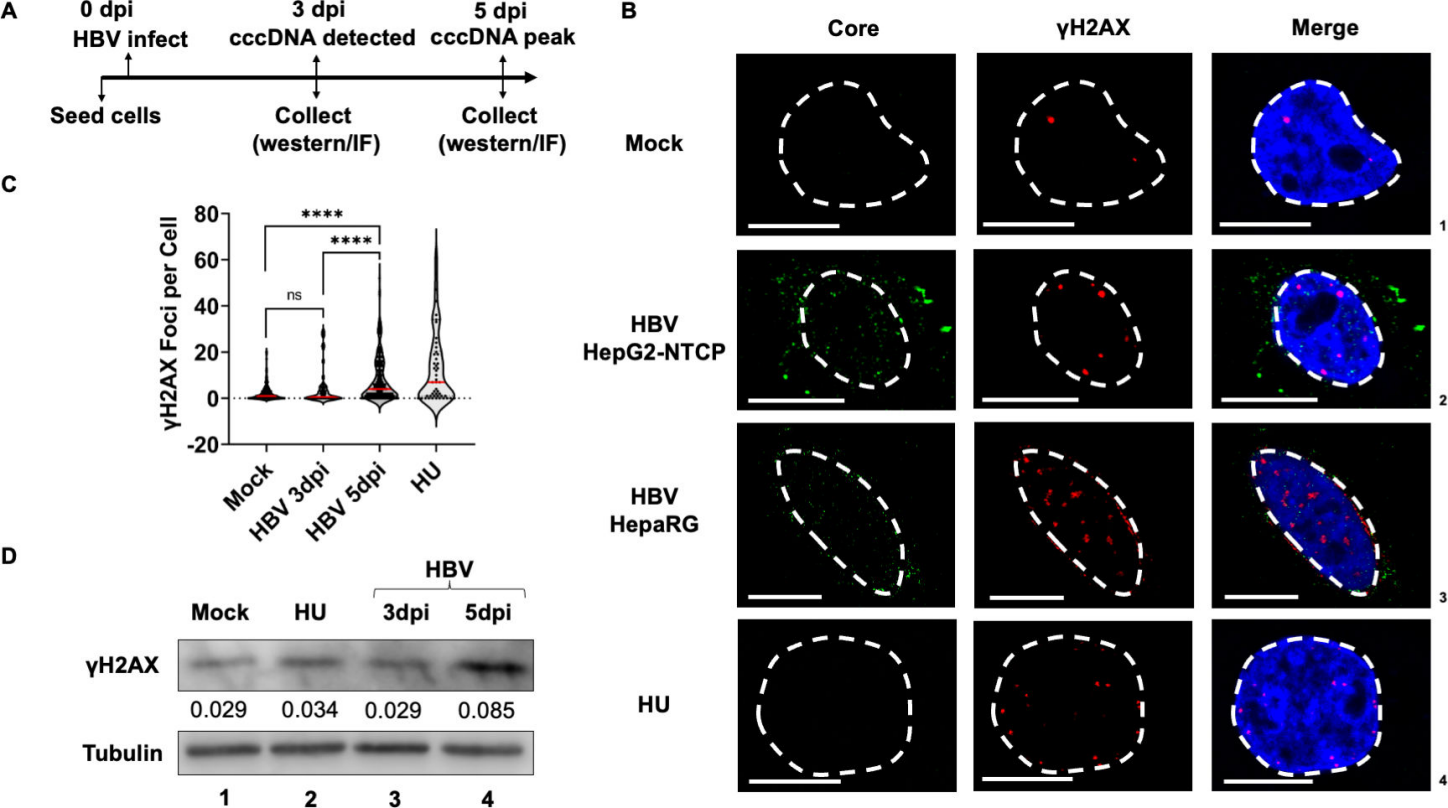
HBV infection provokes DDR signals by Day 5. (**A**) Schematic of plating of HepG2-NTCP cells, HBV infection by spinoculation, and processing for DNA damage by immunofluorescence. (**B**) Representative images of DDR induction in HBV-infected HepG2-NTCP cells (rows 1, 2, and 4) and HepaRG cells (row 3) monitored by staining for the assembled core protein (green) assessed by γH2AX staining (red). The nuclei are marked by DAPI staining (blue), nuclear borders demarcated by dashed white lines, and scale bars in the representative images represent 10 micrometers. HepG2-NTCP cells pulsed with hydroxyurea (HU) for 12 hours prior to processing for immunofluorescence were used as positive control for γH2AX staining. Data are representative of three independent experiments of independent infections. (**C**) Nuclei in multiple HBV-infected nuclei (presented in 1B) were measured during independent viral infections at 3 dpi and 5 dpi. Infected cells were identified by staining for HBV core protein, and the number of γH2AX foci was counted in at least three independent replicates of viral infection. The red bar represents median values with statistical analysis performed using the Mann-Whitney test. Statistical significance is represented by ****, *P* < 0.00005. ns represents nonsignificant difference in between the treatment conditions. (**D**) Immunoblot analysis of HBV-infected HepG2-NTCP cells at the indicated timepoints compared with mock-infected and HU treated cells monitored for γH2AX levels and tubulin levels as loading control. The densitometry values of γH2AX levels are presented by the numbers in the middle.

To determine how HBV infection impacts host genome stability, we performed immunofluorescence analysis in HepG2-NTCP cells infected with HBV at 20 genome equivalents (GEQ) for 3 and 5 days (schematized in Fig. 1A). It has previously been shown that HBV rcDNA is converted into cccDNA molecules that increase in copy number from 1 day post-infection (1 dpi) (56, 57) to 3 dpi (57), which we have independently corroborated, further observing that cccDNA levels plateau at 5 dpi (Fig. S1). To investigate the impact of HBV infection on cellular DDR signals measured by phosphorylated H2AX (γH2AX) (58) levels at single-cell resolution, we performed confocal imaging for γH2AX foci in HBV-infected HepG2-NTCP cells that were co-stained for HBV core protein to identify virus-infected cells. HBV-infected cells at 3 dpi had a median number of 0.5 γH2AX foci (Fig. 1C). However, at 5 dpi, HBV-infected cells had a median number of 6.0 γH2AX foci. To independently verify these observations in an additional HBV-permissive cell line, we performed γH2AX imaging assays in HepaRG cells infected with HBV at 20 GEQ for 5 days. HepaRG cells accrued more γH2AX foci than HepG2-NTCP cells (Fig. 1B, row 3). It has previously been shown that cccDNA production in HepG2-NTCP cells is initiated at 3 dpi and plateaus at 5 dpi (56, 57), which we confirmed using cinqPCR (59) (Fig. S1). We monitored the impact of long-term HBV infection on the cellular DDR by measuring the levels of γH2AX in whole-cell lysates at 5 dpi. As shown in Fig. 1D, HBV infection led to an increase in total nuclear γH2AX at 5 dpi, which was three-fold higher than that of γH2AX at 3 dpi. Importantly, the cellular γH2AX levels at 3 dpi were equivalent to that of Mock-infected cells, suggesting there is no detectable DDRs in HBV-infected cells at this time point. This suggested that conversion of rcDNA to cccDNA correlates with induction of cellular DDR.

### HBV infection induces replication stress on the host genome

**Fig 2:**
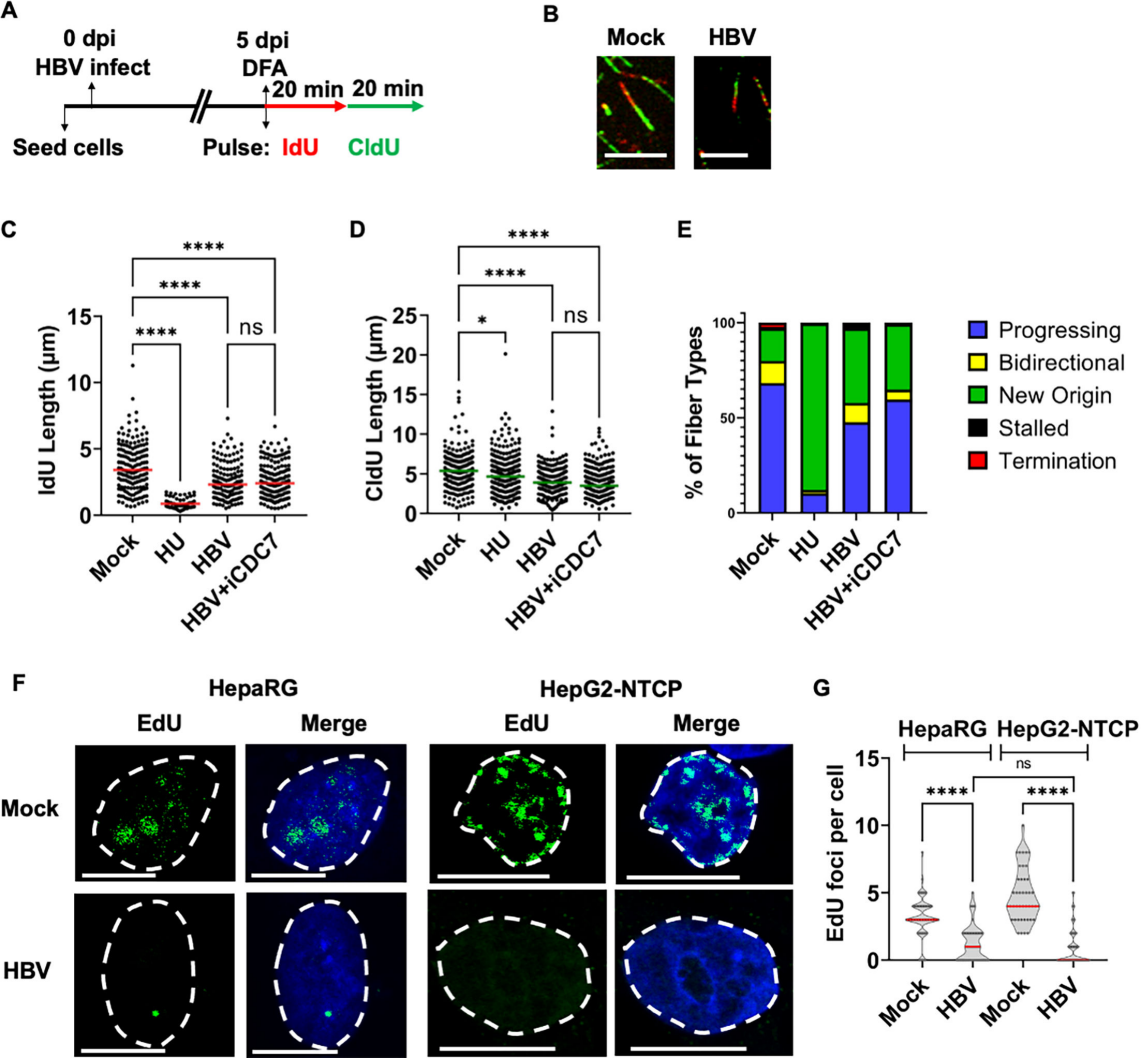
HBV infection induces replication stress on the host genome. (**A**) Schematic of HBV infection of HepG2-NTCP cells followed by sequential pulses of IdU and CldU prior to processing for DNA fiber analysis. (**B**) Representative fibers of HBV-infected HepG2-NTCP cells at 5 dpi showing IdU (red) and CldU (green) incorporation. Scale bars represent 5 micrometers. (**C, D**) Individual fiber lengths were measured by quantifying the IdU and CldU lengths in HBV-infected cells. Each datapoint represents the length of a single IdU- and/or CldU-labeled DNA fiber in HepG2-NTCP cells. The experiment was performed as described in the schematic described in panel A. At least 150 individual DNA fibers were measured for each condition across at least two independent infections. The horizontal lines (red for 2C and green for 2D) represent median values of all datapoints. Statistical analysis was performed using Mann-Whitney test, with **** representing *P* < 0.0005 and * representing *P* < 0.05. (**E**) Categorization of DNA fiber types as percentages of total of 100%, as determined by the presence of IdU or CldU that were divided into percentages that are progressing, bidirectional, stalled, terminated replication forks, and new origin firings in HBV-infected HepG2-NTCP cells. (**F**) Representative images of HepaRG cells (left panels) and HepG2-NTCP cells (right panels) labeled with EdU to indicate nascent DNA (green) in the absence (top panels) and presence of HBV infection (bottom panels) at 5 dpi. Blue represents DAPI staining. Nuclear borders are demarcated by dashed white lines, and scale bars represent 10 microns. (**G**) The number of EdU foci per nucleus was measured in two independent infections, and the median numbers of foci per nucleus are represented in violin plots. Red bars indicate the median number of foci across at least 30 nuclei per treatment. Statistical analysis was performed using the Mann-Whitney test, with **** representing *P* < 0.0005 and ns designating nonsignificant statistical difference.

To determine whether cellular replication stress is associated with host DDR signals, we examined the impact of HBV infection on host replication forks using single-molecule DNA fiber assay (DFA, schematized in Fig. 2A) (60). Briefly, DFA utilizes sequential pulsing of BrdU analogs chlorodeoxyuridine (CldU) and iododeoxyuridine (IdU) to measure how cellular replication forks are impacted upon the introduction of genotoxic stress (60). HBV infection at 5 dpi led to shortening of cellular replication forks (representative examples shown in Fig. 2B), measured by the length of IdU tracks (Fig. 2C) and CldU tracks (Fig. 2D). The median length of IdU-labeled cellular tracks decreased from 3.42 µm in mock-infected cells to 2.32 µm in the presence of HBV, while that of the CldU-labeled tracks decreased from 5.40 µm in mock cells to 3.90 µm in HBV-infected cells. Notably, the mock-infected cells in these experiments were spinfected with cell culture media from Hep-AD38 cells, where HBV production is suppressed with doxycycline treatment (+Dox), demonstrating that cellular components associated with the cultures of HBV producer cells are not sufficient to induce fork stalling. Categorization of the host DNA fibers revealed an increase in forks containing new origin firings (Fig. 2E, green fraction) and a decrease in progressing replication forks in HBV-infected cells compared with uninfected cells (Fig. 2E, blue fraction). These findings suggested that HBV infection may induce aberrant firing of new origins in the host cell.

Replication stress on the eukaryotic genome is regulated by phosphorylation of the Minichromosome Maintenance (MCM) helicase complex (61). Interestingly, inhibition of MCM helicase phosphorylation using the CDC7 inhibitor PHA767491 partially decreased new-origin firing (Fig. 2E, green fraction) without impacting the median shortening of replication forks in HBV-infected cells (Fig. 2C and D), suggesting that HBV-induced replication stress is independent of MCM helicase activity.

To independently confirm our observations made with DFAs, we visualized nascent DNA formation at the single-cell level in two distinct cell lines that are susceptible to HBV infection: HepaRG cells (Fig. 2F, left panels) and HepG2-NTCP cells (Fig. 2F, right panels). At day 5 post-infection, we pulsed these cells with 5-ethynyl 2’-deoxyuridine (EdU) for 2 hours to fluorescently label the newly synthesized DNA segments with an Alexa Fluor 488-conjugated dye using a Click-iT reaction. Uninfected HepaRG cells were measured to contain a median number of three foci per cell, whereas HBV-infected cells had a median of one focus per cell (Fig. 2G). HepG2-NTCP cells have a median of 4 EdU foci per cell, which decreases to zero at 5 dpi (Fig. 2G). It is important to note, however, that despite the changes in the median number of foci, these are a population of nonsynchronous cells, some of which have more foci than the median number. These cells might account for the increased DNA fiber lengths observed in the distribution of IdU/CldU labels in DFAs of HBV-infected cells (described above). Taken together, the DFA and EdU-pulsing studies suggested that HBV infection attenuates replication fork progression, concurrent with the DDR induction that we previously observed using immunofluorescence analysis (Fig. 1B and C).

### HBV triggers cellular replication stress early in infection

**Fig 3:**
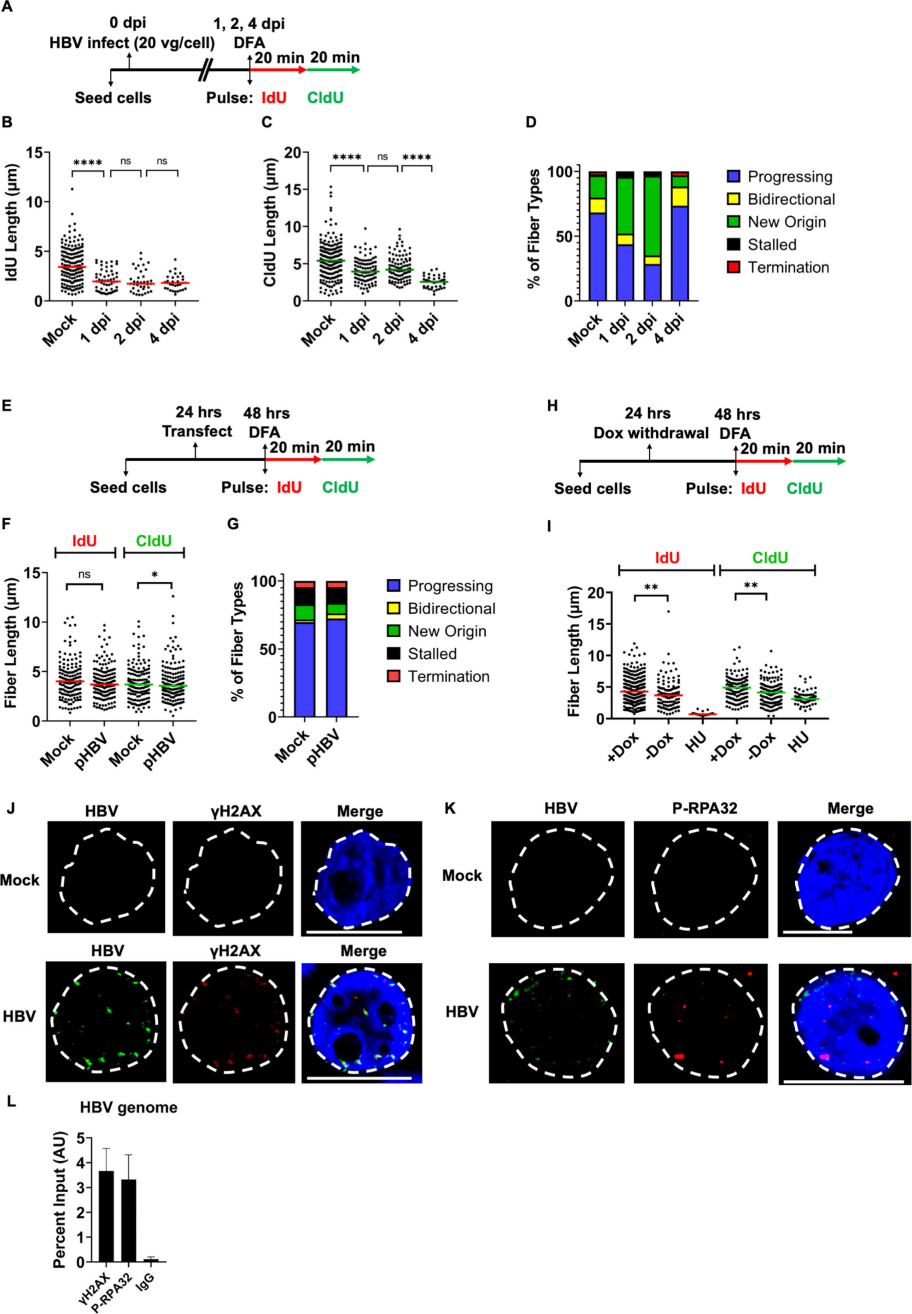
HBV triggers cellular replication stress early in infection. (**A**) Schematic of HBV infection of HepG2-NTCP cells at different time points followed by IdU/CldU pulsing for 20 minutes each before processing for DNA fiber analysis. (**B, C**) Individual fiber lengths were measured by quantifying the IdU and CldU lengths in HBV-infected cells at the indicated time points post-infection. Each datapoint represents the length of a single IdU- and/or CldU-labeled DNA fiber in HepG2-NTCP cells. The experiment was performed as described in the schematic described in Fig. 3A. At least 150 individual DNA fibers were measured for each condition across at least two independent infections. The horizontal lines (red for 3B and green for 3C) represent median values of all datapoints. Statistical analysis was performed using the Mann-Whitney test, with **** representing *P* < 0.0005 and * representing *P* < 0.05. (**D**) Categorization of DNA fiber types as percentages of total of 100% as determined by presence of IdU or CldU that were divided into percentages that are progressing, bidirectional, stalled, terminated replication forks, and new origin firings in HBV-infected HepG2-NTCP cells at the indicated time points post-infection (60, 61). (**E**) Schematic of transfection of HBV infectious clone plasmids in HepG2 cells for 24 hours prior to IdU/CldU pulsing for DNA fiber analysis. (**F**) Individual fiber lengths were measured for the IdU (length) and CldU (right) labels in pHBV-transfected cells at 24 hours post-transfection, as described above. At least 150 individual DNA fibers were measured for each condition across at least two independent transfections. The horizontal lines (red for IdU on left and green for CldU on right) represent median values of all datapoints. Statistical analysis was performed using the Mann-Whitney test, with **** representing *P* < 0.0005 and * representing *P* < 0.05. (**G**) Categorization of DNA fiber types as percentages of total of 100%, as determined by presence of IdU or CldU that were divided into percentages that are progressing, bidirectional, stalled, terminated replication forks, and new origin firings in HBV infectious clone-transfected HepG2 cells at 24 hours post-infection. (**H**) Schematic of DNA fiber analysis in HepAD38 cells at 24 hours post withdrawal from doxycycline. The cells were pulsed with IdU and CldU for 20 minutes each prior to DNA fiber analysis, as described above. (**I**) The individual fiber lengths for the treatments are shown for IdU lengths (left) and CldU lengths (right), with horizontal lines representing median values. Statistical analysis was performed using Mann-Whitney test, with ** representing *P* < 0.01. (**J, K**) Representative images of the HBV genome (green) localizing with cellular sites containing the following: (**J**) DNA damage marker (γH2AX, red) and (**K**) replication stress marker (P-RPA32, red). The nuclei are marked by DAPI staining (blue), nuclear borders are demarcated by dashed white lines, and the scale represents 10 micrometers. (**L**) ChIP-qPCR assays of γH2AX and P-RPA32 binding to HBV genomes at 5 dpi in HepG2-NTCP cells monitored at the viral Cp promoter. Data are represented as mean ± SEM of percent input pulldowns from three independent experiments. IgG pulldowns serve as the negative control.

The correlation between HBV-induced cellular DDR and observation of cellular replication stress at 5 dpi led us to ask when HBV induces the shortening of cellular replication forks. To determine whether HBV-induced replication stress is a cause or consequence of cellular DDR signals, we performed DFA at daily time points post-infection (Fig. 3A through D). Host replication fibers were shortened within 1 day post-infection (1 dpi), which persisted at the same level for 2 days at the level of IdU and CldU fiber lengths (Fig. 3B and C). As infection progressed to 4 dpi, the CldU lengths decreased substantially to 2.556 mm. Characterization of replication events at these time points revealed a progressive decrease in the proportion of progressing forks upon HBV infection at 1 dpi, which further decreased at 2 dpi (Fig. 3D). Next, we asked whether the plasmid form of the viral genome was sufficient to induce replication stress. We transiently transfected HepG2 cells with the HBV-encoding plasmid TMA153 that expresses pgRNA from a CMV-driven promoter (TMA153 is herein referred to as pHBV [62]) and performed DFA 24 hours post-transfection (Fig. 3E through G). When compared to cells transfected with an empty vector control, pHBV did not induce substantial cellular replication stress, as measured by IdU levels and a minor decrease in median replication fork lengths by CldU lengths (Fig. 3F). pHBV additionally did not alter the fractions of replication events (Fig. 3G), suggesting that the expression of viral proteins from the plasmid-based HBV genome is insufficient to induce significant cellular replication stress. However, we note that pHBV transfection heterogeneously introduces the plasmid at different efficiencies in the cell population, which can make the results of pHBV-based DNA fiber assays difficult to interpret. We concluded from these studies that viral entry mechanisms are needed to destabilize cellular replication forks robustly.

HBV-induced replication stress observed at 1 dpi raised the possibility that in addition to entry processes, early replication events might induce replication stress. To determine whether the early stages of HBV expression and replication are sufficient to induce replication fork aberrations, we performed DFAs in HepAD38 cells (using the schema shown in Fig. 3H) that carry a stably integrated copy of the HBV genome under control of a tetracycline-off promoter (63). As shown in Fig. 3I, HepAD38 cells where HBV replication is arrested with doxycycline had longer replication forks than those without doxycycline. Together, these findings suggested that viral replication at the earliest stages is sufficient to induce cellular replication stress. We predict that this replication stress at early stages leads to DDR signals at late stages (5 dpi or after) of infection.

The timing of HBV genome processing events correlating with induction of replication stress and cellular DDR induction seemed to mirror that of DNA viruses like HPV (47, 64), MVM (49), and EBV (50, 65), where the viral genome is in spatial proximity to the cellular DDR markers. Interestingly, HBV genomes in HepG2-NTCP cells also localized closely with cellular DDR markers monitored by γH2AX (Fig. 3J, lower panel) and replication stress markers like RPA32 phosphorylated at Serine 8 (P-RPA32, Fig. 3K, lower panel). To independently confirm the spatial proximity of cellular DDR and replication-stress proteins with the HBV genome, we performed ChIP-qPCR for these proteins on the HBV genome. As shown in Fig. 3L, both γH2AX and P-RPA32 pulldowns yielded positive signals for the HBV genome when monitored using qPCR assays for the Cp promoter. However, it remains unknown whether these factors are directly bound to the HBV genome or detected by secondary crosslinking to cellular DDR sites, where the HBV genome might associate, which remains a technical limitation of imaging assays. Importantly, it is also difficult to determine using these imaging and qPCR assays whether potential DDR signals at these cellular sites are caused by HBV infection. These observations made it critical to interrogate the localization of HBV genomes in an unbiased manner.

### HBV genomes associate with cellular sites enriched in DDIT3 binding elements

**Fig 4:**
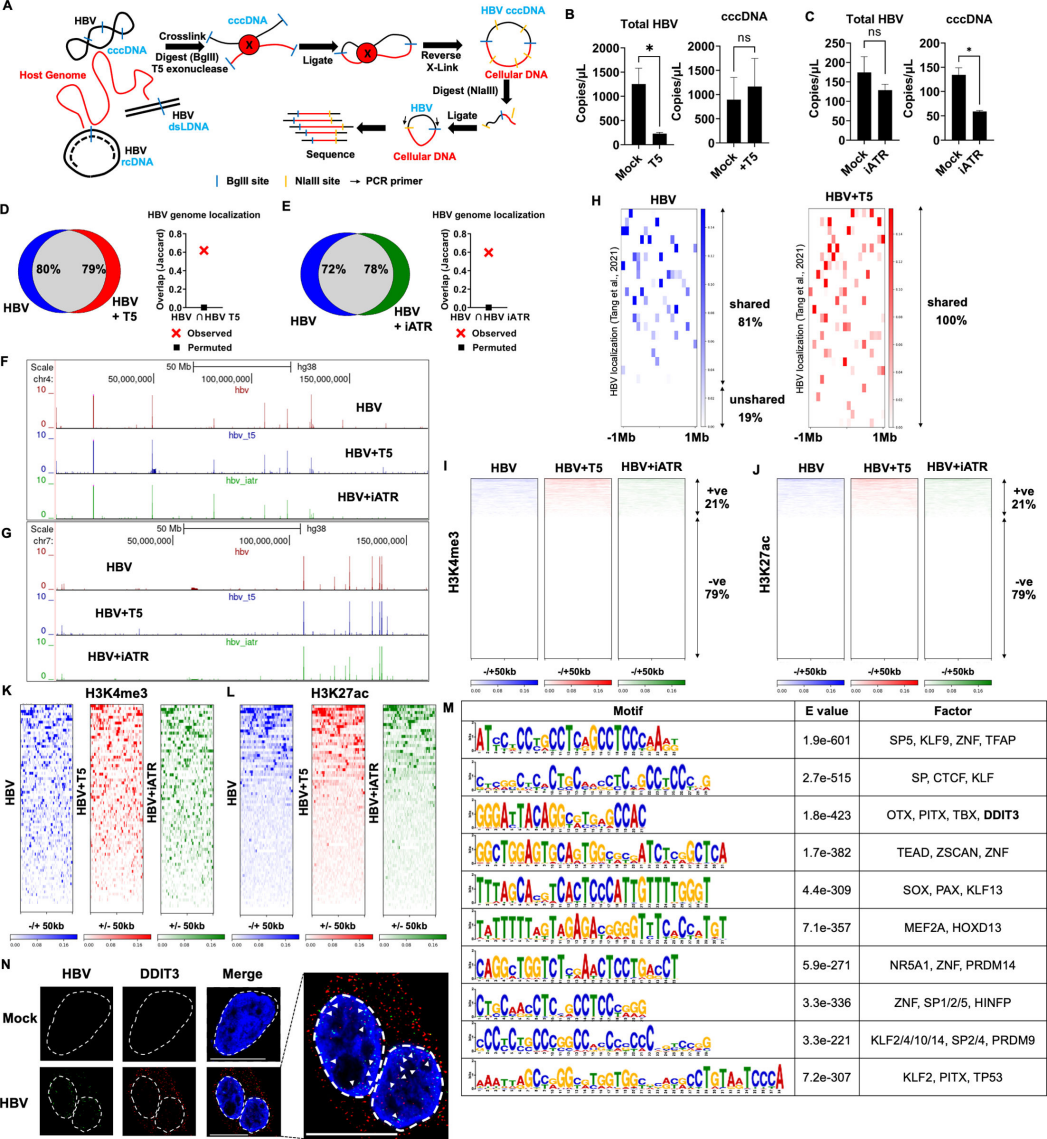
HBV genomes associate with cellular sites enriched in DDIT3 binding elements. (**A**) Schematic of viral chromosome conformation capture assay that incorporates T5 exonuclease treatment to capture the localization sites of HBV cccDNA molecules on the human genome in HepG2-NTCP cells. (**B, C**) Quantification of total HBV DNA (left) monitored by qPCR and cccDNA molecules (right) monitored by cinqPCR analysis in the presence of (**B**) T5 treatment and (**C**) in the presence of ATR inhibitors. Statistical analysis is represented by unpaired Student’s *t*-test with * representing *P*-value < 0.05 and ns designating nonsignificant statistical difference. (**D**) Venn diagram (left) comparing the total genomic regions associated with all HBV genomic forms (blue circle) compared with those sites that are associated with HBV cccDNA molecules (red region) with the overlap indicated by the gray circle. Statistical analysis of total HBV localization relative to that of cccDNA molecules is permuted in the right-hand side using Jaccard analysis, where the intersection is represented by red crosses (Observed). The control intersection was calculated by Jaccard analysis, where cccDNA-associated sites were intersected with a randomly generated library of genomic sites of the same size, and number as total HBV localization, which is indicated by a black square (Permuted). Jaccard analysis values range from 0 to 1, with 0 indicating no intersection and 1 indicating complete intersection. (**E**) Intersection of total HBV genome-associated forms with that of genomic sites where HBV genomes localize in the presence of ATR inhibitor (left). The statistical significance of the overlap is presented as Jaccard analysis on the right-hand side. (**F, G**) Representative UCSC genome browser plots on human chromosome 4 (**F**) and chromosome 7 (**G**) comparing where all HBV genome forms localize (top), HBV cccDNA localization (middle), and HBV genome localization in the presence of the ATR inhibitor (bottom). The x-axis represents the distance along the indicated human chromosome, and y axis represents sequencing reads. (**H**) The locations of HBV-associated genomic sites in this study were compared with previously published HBV localization sites using the Deeptools program on the Galaxy project analysis platform. Heatmaps were generated interrogating where HBV localization is detected relative to 1 Megabase windows of previously identified HBV localization sites. Regions containing positive signals in the heatmap were designated as conserved between the studies. (**I, J**) HepG2 chromatin profiling data for the chromatin marks (**I**) H3K4me3 and (**J**) H3K27ac were interrogated for the presence of HBV genome localization within the 100 kb windows. As a corollary, (**K, L**) HBV-associated genomic sites were interrogated for the presence of (**K**) H3K4me3 and (**L**) H3K27ac within the 100 kb window. Presence of strong blue, red, or green signals in the heatmaps indicated positivity. (**M**) MEME and TOMTOM analysis platforms were used to compute the over-represented motifs and their corresponding transcription factor-binding sites on the human genome that are associated with cccDNA molecules (identified by T5 treatment). The statistical significance of the identified transcription factors is represented by the respective E values. (**N**) Immuno-FISH validation of the HBV genome (red) relative to that of the cellular transcription factor DDIT3 (green) was visualized in HBV-infected (bottom) HepG2-NTCP cells compared with mock-infected cells (top) at 5 dpi. The right panel denotes an enlarged version of the bottom right panel with HBV-DDIT3 foci that are spatially close indicated by white arrows. The nuclei were visualized by DAPI staining, nuclear borders are demarcated by white dashed lines, and the horizontal scale bar represents 10 micrometers.

Previous work has associated HBV genomes with cellular sites that are packaged in transcriptionally active chromatin (also known as type A) chromatin (66). Other DNA viruses, like human papillomavirus and parvoviruses, have shown similar properties (48, 49). However, the generation of multiple extrachromosomal forms of the viral genome during replication in the host, such as rcDNA, double-stranded linear DNA (dslDNA,) and cccDNA (57), makes it challenging to track which genomic forms associate with which cellular sites. To identify sites of association of cccDNA molecules on the host genome, we have incorporated T5 exonuclease treatments into the high-throughput sequencing pipeline (schematized in Fig. 4A) that degrade the dslDNA and rcDNA forms of HBV. This scaled-down assay, which we dub V3C-T5-seq, has enabled us to track the nuclear localization of cccDNA relative to the host genome in a significantly smaller number of cells (600,000 versus 10 million required by traditional chromosome conformation capture assays). We have performed V3C-T5-seq assays with BglII as the primary restriction enzyme, which captures the localization of 73% of the HBV genome relative to the host in a fragment that contains all four HBV promoters: Cp, X-promoter, pre-S1, and pre-S2 (Fig. S2). By retaining the cccDNA molecules in the nucleus (Fig. 4B), V3C-T5-seq has enabled us to map the nuclear location of the cccDNA reservoirs. We have compared these localization sites with HBV-infected HepG2-NTCP cells that are treated with the ATR inhibitor berzosertib that reduces the conversion of rcDNA into cccDNA molecules by 2.5-fold (Fig. 4C). Genome-wide analysis of two independent replicates of HBV chromosome conformation capture analyses revealed that 80% of all HBV-associated genomic sites overlap with cccDNA-specific nuclear sites (Fig. 4D). Interestingly, 78% of the rcDNA-associated genomic sites also overlapped with the total HBV-associated genomic regions (Fig. 4E). Genome-wide visualization of the HBV genome localization peaks revealed a surprising correlation between samples that were processed without treatment (labeled as HBV, Fig. 4F and G; Fig. S3), treated with T5 to retain the cccDNA molecules (HBV+T5, Fig. 4; Fig. S3) and those treated with iATR to inhibit cccDNA formation (HBV+iATR, Fig. 4; Fig. S3). This suggested that there are bonafide cellular sites where all HBV genomic forms associate and persist.

As described above, previous studies have mapped the genome-wide localization of HBV genomes in large populations of 10 million hepatocytes, discovering that HBV associates with discrete chromatin domains packaged in Type A chromatin (53, 66). To determine how the HBV-associated genomic sites identified by V3C-seq and V3C-T5-seq correlated with previously identified HBV-localization sites (53), we overlapped our findings with the previously identified chromatin domains associated with HBV. Almost 81% of the HBV-localization sites identified by our experiments were within the 2-megabase window of previously identified HBV-associated domains (Fig. 4H, left). Strikingly, 100% of our identified HBV cccDNA localization sites were within the 2 megabase windows of the previously identified HBV localization sites (Fig. 4H, right). These observations showed that our V3C-seq studies are consistent with previously published findings, extending them to sharper peaks, likely due to the smaller cell numbers interrogated in our studies. To elucidate how HBV-associated genomic sites correlate with active host chromatin marks, we intersected the findings of our chromosome conformation capture studies with chromatin profiling data in HepG2 cells from the ENCODE project (67). Investigation of genome-wide ChIP-seq peaks for the active promoter-associated chromatin mark H3K4me3 (histone H3 trimethylated at lysine 4, Fig. 4I) and active chromatin mark H3K27ac (histone H3 at acetylated lysine 27, Fig. 4J) revealed that 21% of all host genomic sites packaged in these marks correlated with HBV localization within 100 kilobase domains of the posttranslational modification. This observation suggested that active chromatin was not sufficient to facilitate HBV genome localization (of any form). Conversely, we interrogated the location of the H3K4me3 and H3K27ac ChIP seq peaks relative to that of the HBV localization sites. Surprisingly, these studies showed that HBV localization sites contained strong peaks associated with transcriptionally active promoters (H3K4me3) within the 100 kb genomic region spanning virus-associated sites (Fig. 4K, see strong blue, red, and green data points). However, approximately 50% of the HBV localization sites were associated with the active chromatin mark H3K27ac (Fig. 4L, see diminished blue, red, and green data points in the bottom half of the heatmaps). These findings suggested that HBV-associated genomic sites are likely to exist in the vicinity of transcriptionally active promoters.

To determine which host consensus motifs and transcription factors might be enriched at the HBV-associated genomic sites, we performed *in silico* analysis of the highest 37 V3C-T5-seq peaks using the MEME and TOMTOM bioinformatics suites (68). The enriched motifs associated with cccDNA molecules are represented in Fig. 4M, and the statistical significance of these findings is represented in the middle column. These consensus sequences were associated with host-cell transcription factor-binding sites, indicated in the right column (Fig. 4M). Several of these host factors are associated with regulating the HBV life cycle, such as CTCF (69), while others are associated with oncogenic progression, including TP53 (70), KLF13 (71), SP5 (72), and TBX (73). Among these factors, DDIT3, also known as CHOP (C/EBP homologous protein), has previously been shown to be associated with HBV-induced liver cancers (55). Excitingly, the DDIT3 protein colocalized with the HBV genome in HBV-infected HepG2-NTCP cells at 5 dpi (Fig. 4N, lower panel and enlarged in right panel), demonstrating proof-of-concept for our analytical pipeline connecting HBV localization to consensus sequences on the host genome. Taken together, these findings suggested that the HBV genomes localize to actively transcribing promoter elements that are enriched in DDIT3-binding elements.

### DDIT3 knockdown rescues HBV-induced replication stress

**Fig 5:**
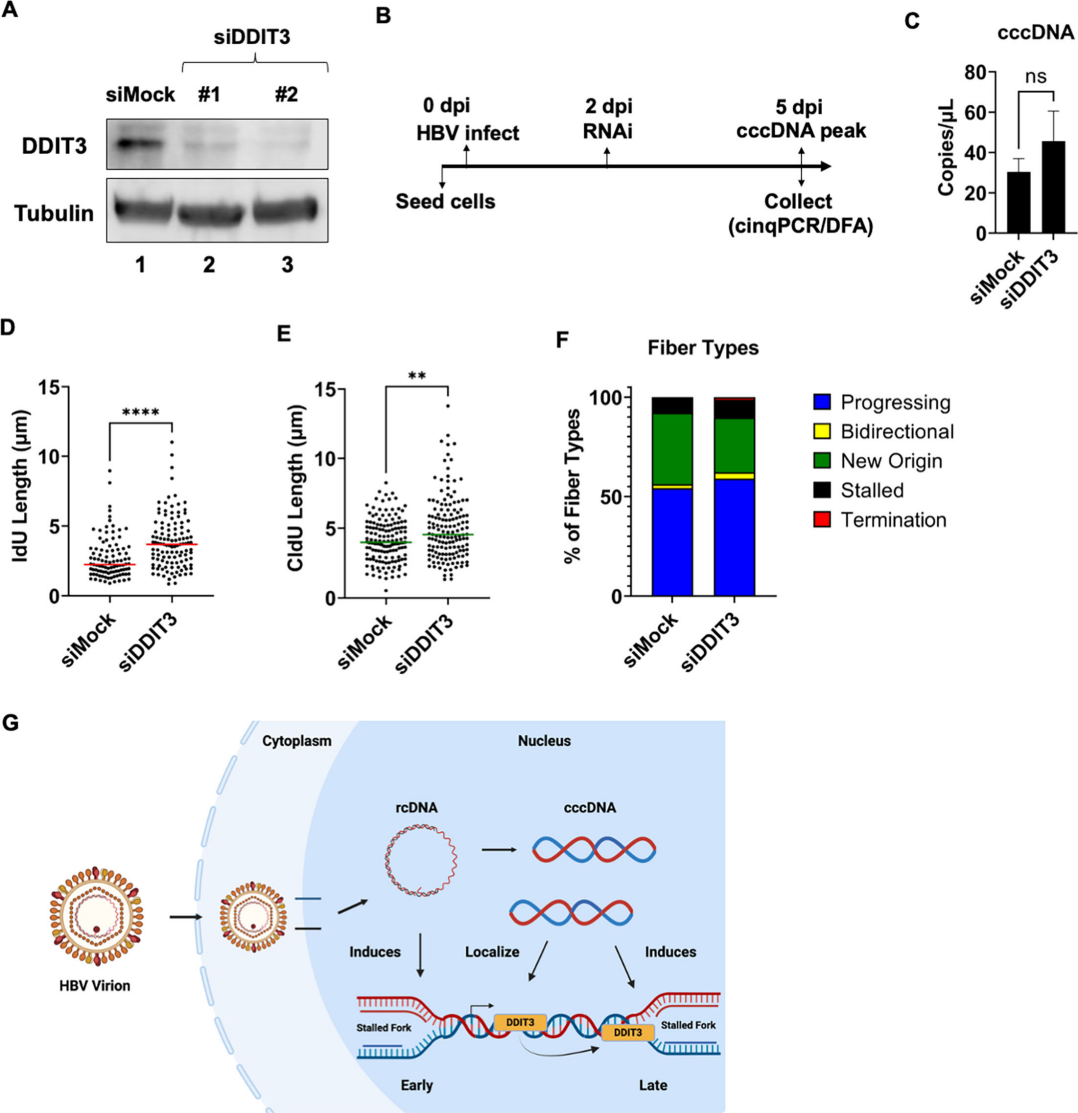
DDIT3 knockdown rescues HBV-induced replication stress. (**A**) DDIT3 immunoblots in HepG2-NTCP cells transfected with two RNAi constructs to evaluate the effectiveness of knockdown efficiency (compared with siMock knockdown control). (**B**) Schematic of DDIT3 silencing during HBV infection of HepG2-NTCP cells to evaluate the impact on (**C**) cellular cccDNA reservoirs and (**D–F**) induction of host-cell replication stress. Individual fiber lengths were measured by quantifying the IdU and CldU lengths in HBV-infected HepG2-NTCP cells at 5 days post-infection. At least 150 individual DNA fibers were measured for each condition across three independent infections. The horizontal lines (red for 5D and green for 5E) represent median values of all datapoints. Statistical analysis was performed using the Mann-Whitney test, with **** representing *P* < 0.0005 and ** representing *P* < 0.01. (**F**) Categorization of DNA fiber types as percentages of total of 100%, as determined by the presence of IdU or CldU that were divided into percentages that are progressing, bidirectional, stalled, terminated replication forks, and new origin firings. (**G**) Mechanism of HBV-induced replication stress that leads to genome instability and the contribution of cccDNA localization sites in disrupting replication dynamics.

To determine how DDIT3 regulates HBV lifecycle, we performed RNAi-mediated knockdowns in HepG2-NTCP cells. As shown in Fig. 5A, we tested two different siRNAs’ targeting DDIT3 transcripts, discovering that siRNA number 2 was more effective at depleting DDIT3 protein levels (Fig. 5A, lane 3). We transfected this siRNA into HBV-infected HepG2-NTCP cells at 2 days post-infection, harvesting the cells at 5 dpi to assess the impact on cccDNA formation and impact on host replication forks by DFAs (Fig. 5B). While depletion of cellular DDIT3 did not impact the HBV cccDNA levels (Fig. 5C), we observed that the absence of DDIT3 led to a rescue of HBV-induced fork shortening at 5 dpi. This rescue of HBV-induced replication stress was evident at the level of IdU track lengths (Fig. 5D) and CldU track lengths (Fig. 5E) to comparable levels in mock-infected cells. These changes in fiber lengths were associated with an increase in the proportion of progressing replication forks that were detectable in these cells (Fig. 5F). Taken together, our studies indicate that HBV genomes associate with cellular sites of transcriptionally active promoters where host factors like DDIT3 contribute to HBV-induced replication stress that leads to eventual cellular DNA breaks.

## DISCUSSION

In this study, we have explored the connection between HBV genome localization and the cellular DNA damage response. HBV infection is sufficient to induce cellular replication stress early. This replication stress is likely caused by a combination of several factors, including perturbed entry pathways, genome processing, and expression of early viral proteins (Fig. 5G). While the cause of these replication stress signals remains largely unknown, they manifest in cellular DDRs only at late stages of infection (5 days in HepG2-NTCP cells, after the establishment of a significant latent viral reservoir). The viral genome localizes to, and persists, in the vicinity of transcriptionally active promoter regions, perhaps exacerbating replicative stress through transcription-replication conflicts on the hepatocyte genome. Importantly, a significant number of these HBV localization sites are associated with the stress-induced transcription factor DDIT3. Since the absence of DDIT3 rescues HBV-induced replication stress, these observations suggest that HBV genomes exacerbate cellular DDRs through DDIT3-dependent mechanisms (Fig. 5G). Taken together, our observations suggest the existence of a connection between HBV genome location and host genome stability. In primary cells, this might regulate oncogenic progression that leads to hepatocellular carcinomas, a topic of future studies.

Upon entry, DNA viruses localize to distinct nuclear sites that sustain the viral life cycle through gene expression, replication, and persistence (74). These nuclear sites are rich in replication proteins, chromatin modifiers, and transcription factors (10). These cytological observations have been collectively referred to as promyelocytic leukemia (PML) bodies, which also colocalize with HBV genomes and proteins during replication (75–77). Our findings in this study add to this catalog of HBV-associated cellular factors by including proteins in the DNA damage response and replication stress response pathways. However, it remains unclear whether DDR factors that colocalize with HBV are directly bound to the viral genome, associate with viral proteins, or are bound to the cellular sites in the vicinity of the virus. While DDR proteins such as those in the ATR pathway are required to process rcDNA into cccDNA (33), it remains unclear whether they continue to persist on the viral genome long term after conversion. Recent observations that the host MRE11 proteins regulate ATR-mediated formation of cccDNA further suggest that cellular DDR factors play a key role in the HBV life cycle (4). Alternately, it remains conceivable that DDR proteins are the conduits for the recruitment of chromatin modifiers to the viral genome, facilitating the establishment of long-term latency, as has been observed for small DNA viruses such as AAV (78) and HPV (79, 80). Since AAV and HPV have been known to occasionally integrate into the host genome, it remains possible that DDR proteins colocalizing with HBV, as observed in this study, are a result of viral genomes integrating into the host. However, the rarity of HBV integration combined with the high frequency of DDR-HBV colocalization makes this possibility less likely. Interestingly, HBV integration events are at their highest at 5 dpi, correlating with the time point when they accrue DNA damage, although these events are very rare (81). The dissection of how and why DDR proteins associate with HBV, or the host genome in the vicinity of HBV components, warrants further study in synchronous systems of HBV infection.

Studies by Shah and O’Shea in adenoviruses revealed that virus and cellular genomes activate distinct DNA damage responses (82). In support of this model, we have previously discovered that the autonomous parvovirus MVM induces replication stress on the host genome that precedes induction of DNA damage by depleting the host of the single-stranded DNA-binding protein RPA (61). The induction of replication stress by MVM occurs early in the S phase and can be reversed by inhibiting the function of the replication fork helicase CDC7 (61). AAV genomes similarly induce cellular replication stress via RPA exhaustion that represses viral gene expression (83). In contrast, our current study revealed that HBV-induced replication stress is observable within 1 day post-infection, both in HepG2-NTCP cells where HBV uses viral entry mechanisms to enter the nucleus and in HepAD38 cells where HBV replication is induced by doxycycline withdrawal. This virus-induced replication stress is exacerbated over 4 to 5 days of infection, during which time HepG2-NTCP cells have undergone two rounds of mitosis (84). This suggests that HBV-induced replication stress on the host genome accrues progressively over time, preceding the induction of cellular DNA damage. Interestingly, this replication stress is not sufficient to induce a significant cell-cycle block as S-phase entry is required for the nascent DNA to be visualized using DFAs and EdU labeling. These observations resemble the replication stress phenotype induced by other tumor viruses such as HPV and mouse papillomavirus 1 (MmuPV1), that deploy the viral oncoproteins E6/E7 at active chromatin hubs (14–16) and EBV that induces a hit-and-run mechanism using EBNA1 on the host genome at fragile sites (50).

Chromosome conformation capture technologies have proven to be invaluable tools to monitor where DNA viruses localize in the nucleus in an unbiased and high-throughput manner. Prior studies that have deployed these techniques to investigate HBV genome localization have found that HBV genomes associate with active chromatin sites on the human epigenome (53). We have previously used these technologies to show that parvoviruses localize to cellular DDR sites associated with transcriptionally active and accessible chromatin (49, 85). Similar studies with herpesviruses have found that EBV genomes localize to cellular enhancers that are enriched with the transcription factors ZN770, ZN121, PAX5, PRDM6, and IRF3 (86). The major drawback of these chromosome conformation capture assay systems has been their inability to distinguish between different forms of viral genomes, particularly those generated by DNA viruses in the nuclear compartment of dividing cells and their reliance on large numbers of cells that average out heterogeneity in populations of cells. We have attempted to resolve this issue in the current study by incorporating T5 exonuclease treatment into the chromosome conformation capture assay, allowing us to monitor the localization of cccDNA molecules. We observed that cccDNA molecules make up almost half of the total cellular sites that are measurably associated with HBV genomes. Since cccDNA molecules make up only 5–12 copies of DNA molecules per infected cell nucleus (57), comparison with total HBV localization sites that correlate with cccDNA suggests that there is a small subset of genomic hotspots where HBV genomes converge. Most of these genomic sites are associated with transcriptionally active chromatin. Our assays combining *in silico* prediction and imaging reveal that HBV genomes are associated with cellular sites containing the DDR protein DDIT3. Since DDIT3 (also known as CHOP) has been implicated in oncogenic progression of hepatocytes leading to liver cancer (55), this might be one of the connecting links between HBV-induced DDR, cirrhosis, and oncogenesis. Our Immuno-FISH assays corroborate the possible association between the HBV genome and DDIT3, where we see sites of close localization between both. We hypothesize from these observations that DDIT3-mediated induction of reactive oxygen species (87) might exacerbate the replication stress in the host, which are necessary for the conversion of its rcDNA to cccDNA, further suggested by the rescue of HBV-induced replication stress in the absence of DDIT3. As a final consequence of this action, it is likely that DDIT3 overproduction causes cell death and inflammation, which can stimulate tumor formation when dysregulated.

One of the critical drawbacks of chromosome conformation capture assays is the reliance on high cell numbers to detect significant distal interactions. This leads to an averaging of the detected chromatin organizations that masks differences between individual cells or cell types. To begin to overcome these limitations, we have scaled down the V3-T5-seq assay almost 16-fold, using 600,000 cells rather than 10 million that is typically used by 3C- and 4C-based methods. While the detected localization sites on the cellular genome in our study are still within the vicinity of 4C-based techniques previously published (53), our observations yield significantly greater clarity and resolution. Rather than broad megabase-sized peaks, we observe sharp peaks that cover multiple kilobases. This has enabled us to interrogate for the first time the binding elements that are enriched at HBV genome localization sites. It is noteworthy, however, that the strategies used to monitor genome localization in the present work do not differentiate between incoming viral genomes, progeny viral genomes, or genomes that are actively undergoing processing. Therefore, it remains unknown what function the host genomic sites perform in regulating the fate of HBV genomes.

In conclusion, our observations suggest that HBV-induced replication stress begins early at the onset of infection and persists over time and that this stress eventually induces cellular DDR signals. Interestingly, these DDR events and HBV genomes occupy the same subnuclear territories. Furthermore, HBV genomes associate with cellular sites of transcriptionally active chromatin that are enriched in important cellular transcription factors, such as DDIT3. These findings open new avenues to investigate how the location of HBV genomes and cellular DDR proteins at distinct host nuclear sites regulates genome stability that can contribute to oncogenic transformation.

## MATERIALS AND METHODS

### Cell lines and viral stock preparation

Human hepatocyte HepG2 cells that overexpress the NTCP receptor (HepG2-NTCP [88]) were maintained in Dulbecco’s modified Eagle’s medium/nutrient mixture F-12 (DMEM/F-12, high glucose; Gibco) supplemented with 5% Serum Plus (Sigma Aldrich) and 50 μg/mL penicillin/streptomycin (Gibco). HepG2-NTCP cells were maintained on collagen-coated dishes for optimal growth and morphological characteristics. Tissue culture dishes were overlaid with 50 mg/mL rat tail collagen (BD Biosciences) in 0.02 N acetic acid solution and dried at room temperature for at least 1 hour for coating. The plates were then washed with phosphate-buffered saline (PBS) and used immediately or stored at 4°C overnight until use. For all imaging studies on cells plated on glass coverslips and laser micro-irradiation, collagen coating was used to aid in cell adherence. Cells were cultured in 5% CO_2_ at 37°C.

The human hepatocyte cell line HepAD38 (63) was maintained in Dulbecco’s modified Eagle’s medium/nutrient mixture F-12 (DMEM/F-12, high glucose; Gibco) supplemented with 5% Serum Plus (Sigma Aldrich), 50 μg/mL penicillin/streptomycin (Gibco), 50 mg/mL gentamicin (Gibco), and 1 mg/mL doxycycline for the purposes of repression. HBV stock was produced by culturing HepAD38 cells until 70% confluency. Once confluent, doxycycline was removed, and cells were cultured in 5% CO_2_ at 37°C. The supernatant was collected every 4 days. Viral genome concentrations were calculated using qPCR compared with serial dilutions of known genome copies of the HBV infectious clone plasmid. HBV stock was maintained at −80°C.

The human hepatocyte cell line HepaRG was thawed in HepaRG Thaw, Plate & General Purpose Medium Supplement (Thermo Scientific catalog no. HPRG670), supplemented with 100 mL of William’s E medium and 1 mL GlutaMAX supplement. After 1 day, the medium was changed to the HepaRG Maintenance/Metabolism Medium Supplement (Thermo Scientific catalog no. HPRG620), supplemented with 100 mL of William’s E medium and 1 mL GlutaMAX supplement. HepaRG cells were maintained and spinfected with the maintenance medium mixture.

### Viral infection

HepG2-NTCP cells were seeded into 6-well plates with DMEM/F-12 and incubated in 5% CO_2_ at 37°C for 4 hours. Fresh spinoculum was made (DMEM/F-12 supplemented with 3% Serum Plus, 2% DMSO, 1% NEAA, and 4% PEG 8000). The solution was then filtered sterilized with a 0.22 μm filter (Corning). The medium was switched with Spinoculum medium, and HBV stock was added to 20 GEQ. Plates were then wrapped with parafilm and then centrifuged at 1,000 × *g* at 37°C for 1 hour. Parafilm was removed, and plates were incubated in 5% CO_2_ at 37°C for 5 days. All viral infections carried out in these studies used HBV genotype D.

### Plasmids, siRNAs, and transfections

All transfections were performed with linear polyethyleneimine (PEI) with a molecular weight (MW) of 25,000 (Polysciences). Cells were seeded the day before transfection in culture dishes pretreated with collagen. Transfections were carried out in cells that were 60% to 70% confluent. The total mass of DNA transfected per condition was 1 mg/3.8 cm^2^ culture growth area and adjusted accordingly. The DNA to PEI ratio of 1:3 was used in NA-PEI-Opti-MEM mix. The medium on transfected cells was replaced with fresh prewarmed medium between 6 and 18 hours post-transfection. The TMA153 plasmid referred to in this study as pHBV is the same as the previously published pTMA153 expressing full-length HBV pgRNA, Cp, P, and all three envelope proteins (62). The TMA153 plasmid expresses WT HBV where the pgRNA is expressed from the CMV promoter and other HBV proteins are expressed from their respective endogenous promoters. siRNAs used for DDIT3 knockdown were obtained from Thermo Scientific s3997 (siRNA #1 in Results) and s225792 (siRNA #2 in Results).

### Immuno-FISH assays

Immuno-FISH assays were performed on HepG2-NTCP cells that were cultured and infected as described forimmunofluorescence assays. Approximately 20-bp DNA oligos that were complementary to the HBV genome (GAGGCCTGTATTTCCCTGCTG, CCCTGCGCTGAACATGGAGA, CAGAGTCTAGACTCGTGGTGGA, CCTCTTGTCCTCCAACTTGTCCT, TCTTCTGGACTATCAAGGTATGTTGC, GAACCTCTATGTATCCCTCCTGTTGC, GTGGGCCTCAGCCCGTTTCT, ATGTGGTATTGGGGGCCAAG, CAAAGAGATGGGGTTACTCTCTA, GAAAACTTCCTATTAACAGGCCTATT, TATCCTGCGTTGATGCCTTTGT, CTGAACCTTTACCCCGTTGCC, CTTTTCGGCTCCTCTGCCGA, ACTCTGTTGTCCTATCCCGCA, GAATCCTGCGGACGACCCTTC, GACTCCCCGTCTGTGCCTTC, GCCCAAGGTCTTACATAAGAGGAC, GGGGGAGGAGATTAGGTTAAAGGT, CTCTTGTTCATGTCCTACTGTTCA, GAGTTACTCTCGTTTTTGCCTTCTGAC, GTTCACCTCACCATACTGCAC, GAGACCTAGTAGTCAGTTATGTCAACAC, CAGTTATAGAGTATTTGGTGTCTTTCGGAG, GTTGTTAGACGACGAGGCAGG, CTCAATGTTAGTATTCCTTGGACTCA, CCTAATATACATTTACACCAAGACAT, GCCAGGTTTTATCCAAAGGTTACC, CTATTTACACACTCTATGGAAGGCGG, GATCTACAGCATGGGGCAGAATC, GATTGGGACTTCAATCCCAACA, TTTGGGGTGGAGCCCTCAGG, and CCGCTGTCTCCACCTTTGAGA) were designed using Primer3 (89) and purchased from IDT. Oligos were pooled and labeled with 250 mM amino-11-dUTP (Thermo Scientific) and TdT enzyme (Promega). Labeling reactions were carried out overnight at 37°C and were then terminated by incubating at 70°C for 10 minutes. Labeled oligos were precipitated in isopropanol, washed in 75% ethanol, and dissolved in 15 mL of 0.1 M sodium bicarbonate (pH 8.3). 0.75 mL of 20 mM NHS esters (Thermo Scientific) were conjugated to the aminoallyl-tagged oligos for 2 hours in the dark. The labeled oligos were precipitated with isopropanol, washed in 75% ethanol twice, and dissolved in 50 mL nuclease-free water. The labeled oligos were precipitated in isopropanol, transferred to a PCR clean-up column (Promega, A9282), and centrifuged. Columns were washed twice in 80% pre-chilled ethanol and eluted in 50 mL nuclease-free water. HBV-infected HepG2-NTCP cells were CSK pre-extracted for 3 minutes in CSK buffer, followed by CSK buffer containing Triton X-100. Samples were washed with PBS and fixed in 4% paraformaldehyde for 10 minutes at room temperature. Cells were permeabilized with permeabilization buffer for 10 minutes before being washed in PBS and denatured in 10% formamide solution in 2× SSC for 2 hours at 37°C. Then, 2 mL of the probe was mixed with 40 mL of FISH hybridization buffer, mixed, and added to the surface of the glass slide. The coverslip-containing cells were inverted over the FISH hybridization-probe solution, and edges were sealed with rubber cement. Hybridization was carried out overnight at 42°C. Samples were washed twice in 2× SSC containing 0.1% Triton X-100 for 3 minutes each at 42°C. Samples were washed twice with 2× SSC at 37°C for 3 minutes each. Cells were then immunostained starting with blocking in 3% BSA in PBS, as described above for immunofluorescence assays, mounted on DAPI-containing Fluoromount, and imaged using a confocal microscope. Confocal microscopy was performed using a Leica Stellaris 5 on a Leica Stellaris 5 microscope on 63× oil objective and 2.5× digital zoom. Images were acquired using a 405 nm Diode laser and White Light Laser. Acquisition was carried out at 400 Hz, line average of 2, and 25% laser intensity of WLL. Post-acquisition analysis of the imaging was performed using the Leica software’s Lightning Process function with the DAPI fluoromount mounting medium as the pre-calculated deconvolution settings.

### Immunofluorescence imaging

Cells were fixed with 4% paraformaldehyde for 10 minutes at room temperature and then washed with PBS. 0.1% Triton X-100 was added for 10 minutes to permeabilize the cells. Samples were washed with PBS and blocked for 30 minutes with 3% BSA in PBS. Cells were then incubated at room temperature with the indicated primary antibodies (Table 1) for 1 hour, washed with PBS, and incubated for 30 minutes with the indicated secondary antibodies in 3% BSA. Coverslips were washed with PBS and mounted onto slides with Fluoromount containing DAPI (Southern Biotech). DDR signaling antibody effectiveness in detecting DNA breaks was optimized using mock-infected cells and HepG2-NTCP cells treated with hydroxyurea to induce DNA damage.

**TABLE 1:** Antibodies used in this study

Reagent type	Designation	Reference	Identifier	Species
Antibody	γH2AX	EMD Millipore	05-636	Mouse
Antibody	γH2AX	Abcam	ab11174	Rabbit
Antibody	Tubulin	EMD Millipore	05-829	Mouse
Antibody	Hepatitis B Core	OriGene	AP08118PU-N	Rabbit
Antibody	C/EBP-homologous protein (CHOP)/DDIT3	Cell Signaling	2895	Mouse
Antibody	P-RPA32	Cell Signaling	83745	Rabbit
Antibody	Goat anti-rabbit IgG, Alexa Fluor 488	Invitrogen	A-11034	Rabbit
Antibody	Goat anti-mouse IgG, Alexa Fluor 568	Invitrogen	A-11031	Mouse
Antibody	Anti-rabbit IgG	Cell Signaling	7074	Rabbit
Antibody	Anti-mouse IgG	Cell Signaling	7076	Mouse
Antibody	Normal rabbit IgG	Millipore	12-370	Rabbit

### Chromatin immunoprecipitation followed by qPCR analysis (ChIP-qPCR)

HepG2-NTCP cells infected with genotype D HBV virus were crosslinked in 1% formaldehyde for 10 minutes at room temperature. Then, 0.125 M glycine was used to quench the crosslinking reaction for 5 minutes at room temperature. Cells were lysed on ice for 20 minutes in SDS lysis buffer (1% SDS, 10  mM EDTA, 50  mM Tris-HCl, pH 8, protease inhibitor), cell lysates were sonicated using a Diagenode Bioruptor Pico for 60 cycles (15  s on and 30 s off per cycle), before being incubated overnight at 4°C with the antibodies (Table 1) bound to Protein A Dynabeads (Invitrogen). The pulldowns were washed using low-salt wash (0.01% SDS, 1% Triton X-100, 2 mM EDTA, 20  mM Tris-HCl pH8, 150  mM NaCl), high salt wash (0.01% SDS, 1% Triton X-100, 2  mM EDTA, 20  mM Tris-HCl pH8, 500  mM NaCl), and lithium chloride wash (0.25M LiCl, 1% NP40, 1% DOC, 1 mM EDTA, 10  mM Tris HCl pH8) and twice with TE buffer for 3 minutes each at 4°C. Using SDS elution buffer (1% SDS, 0.1 M sodium bicarbonate), DNA was eluted, and crosslinks were reversed using 0.2M NaCl, Proteinase K (NEB), and incubated at 56°C overnight. The pulldown DNA was purified using a PCR Purification Kit (Qiagen) and eluted in 100 µL of Buffer EB (Qiagen). ChIP DNA was quantified by qPCR analysis (Biorad) under the following conditions: 95°C for 5 min, 95°C for 10 s, and 60°C for 30 s for 50 cycles. HBV genome interaction with the respective molecules was assessed by qPCR assays using primers complementary to the HBV genome. The primer sequences used for ChIP-qPCR on the HBV genome in 5 ′to 3′ orientation are as follows: CTCTTGTTCATGTCCTACTGTTCA (forward primer) and AGCTGAGGCGGTATCTA (reverse primer). The pulldowns were computed relative to input levels.

### DNA fiber analysis

HepG2-NTCP cells were plated onto 6-well plates and infected by spinoculation and incubated for the indicated number of days. At the end of the indicated days, cells were pulsed with 20 mM IdU in complete media for 20 minutes. Samples were washed with PBS and pulsed with 50 mM CldU for 20 minutes in media. Cells were then pelleted for 5 minutes at 5,000 × *g* at room temperature, and the supernatant was discarded. Pellets were resuspended in 150 μL of complete media and stored on ice. Two 2 μL resuspensions were then pipetted onto the top of a positively charged slide at each end. A volume of 7 μL of DNA Lysis buffer was added to each resuspension and pipetted gently four times. Samples were left at room temperature for 5 minutes on an even surface. Slides were then tilted at a gentle angle and left for 15 minutes for solutions to spread across the slide. While waiting, a solution of 3:1 methanol/acetic acid was made in a Coplin staining jar. Slides were added upright to the 3:1 solution and left for 5 minutes to allow the DNA to become fixed. Using a new jar, slides were washed three times in PBS and then denatured in a 2.5 M HCl solution for 1 hr at room temperature. Slides were washed three times with PBS. Laying the slides on a flat surface, 500 μL of 3% BSA was added and left for 30 minutes. After blocking, the cells were stained with Abcam rat anti-BrdU (1:1000) and BD Biosciences mouse anti-BrdU (1:500) at room temperature for 30 minutes, with a coverslip gently laid on the slide. After 30 minutes, the coverslip was quickly removed, and the slides were washed with 0.1% Tween 20 in PBS three times. Samples were stained with anti-rat Alexa Fluor 488 and anti-mouse IgG1 Alexa Fluor 568 (1:1,000) at room temperature for 30 minutes under covered conditions. Samples were washed with 0.1% Tween 20 in PBS three times, and cover slips were affixed to slides using ProLong Gold Antifade Mountant (Thermo Scientific). Fibers were then imaged with a Leica Stellaris confocal microscope using a 63× oil immersion objective lens. Fiber lengths were measured using Digimizer software (MedCalc Software Ltd).

### Western blot

HepG2-NTCP cells were plated in 6-well plates at 200,000 cells. Samples were then spinfected with Spinoculum media at 20 GEQ and left to incubate for 5 days. Cells were scraped off and added to a microcentrifuge tube and centrifuged at 5,000 rpm for 2 minutes at room temperature. Samples were then resuspended in RIPA buffer and incubated on ice for 15 minutes. Samples were then centrifuged at 13,000 rpm for 10 minutes at 4°C. The supernatant was collected, and the protein sample concentration was calculated using a bicinchoninic acid (BCA) assay (Bio-Rad).

### Viral chromosome conformation capture combined with T5 (V3C-T5-seq) assays

V3C-T5-seq assays were performed in 600,000 cells with BglII as the primary restriction enzyme to digest HBV-infected HepG2-NTCP chromatin. The BglII site selected as a viewpoint was on the HBV Core promoter region located at nucleotide 1983 in genotype D. Briefly, samples were crosslinked in 1% formaldehyde for 10 minutes before being quenched in 0.125 M glycine on ice for 5 minutes. Cells were lysed in NP-40 lysis buffer for 10 minutes on ice, before resuspending the nuclei in restriction enzyme buffer (Cutsmart Buffer). Samples were permeabilized in 0.3% SDS for 1 hour on a 37°C shaker, sequestered in 2% Triton X-100 for 1 hour on a 37°C shaker. One microliter of T5 Exonuclease (New England BioLabs) was added to the samples for 1 hour on a 37°C shaker. Samples were inactivated with 8 μL of 0.25 M EDTA before centrifuging at 5,000 RPM for 5 minutes. The supernatant was removed, and the pellet was resuspended in restriction enzyme buffer (Buffer 3.1) and digested overnight in 400 U of BglII enzyme with shaking. Samples were further digested with 300 U of BglII for 4 hours, inactivated at 65°C for 20 minutes in 1% SDS, and sequestered with 1% Triton X-100 for 1 hour at 37°C. Chromatin was resuspended in 1.15× T4 DNA ligase reaction buffer, and intramolecular ligation was carried out at room temperature for 4 hours. Crosslinks were reversed, and proteins were digested with proteinase K and RNAse A and heat treated at 65°C. DNA was purified by phenol:chloroform:isoamyl alcohol extraction, isopropanol precipitation, and a PCR purification kit (Qiagen). 3C DNA was eluted in 200 mL of DNA, secondary digested with NlaIII at 37°C overnight, and circularized with 100 U of T4 DNA ligase in 15 mL ligation reaction. The V3C-seq samples were precipitated by phenol:chloroform:isoamyl alcohol extraction, precipitated in isopropanol, and resuspended in 100 mL of Buffer EB (Qiagen). Inverse PCRs were performed on the BglII-NlaIII fragment on the HBV genome on the Core gene downstream of the Core promoter region using inverse PCR primers tgccttctgacttctttccttcagt and cagtagctccaaattctttataaggg. DNA was diluted 1:100 in TE before being used as templates for nested-inverse primer gacttctttccttcagtacg and tctttataagggtcgatgtc. The PCRs were pooled and purified using the PCR purification kit (Qiagen), and sequencing libraries were prepared using the NEB Ultra Kit. Twelve samples were pooled per run for paired-end sequencing using an Illumina Next-seq 500 sequencer. The complexity of the sequencing reactions was increased by spiking in 25% phiX with the sequencing reactions.

### V3C-T5-seq analysis

High-throughput V3C-seq and V3C-T5-seq studies were aligned to the human hg38 reference genome using single-end sequencing parameters in Minimap2 alignment program (90). Samples were sorted with Samtools (91). Genome-wide coverage of the aligned intervals was measured with BEDtools (92). Independent biological replicates of the high-throughput sequencing studies were merged using BEDtools (92). Comparative analysis with published ChIP-seq and fragile site sequencing studies was performed using the Deeptools package (93) on the Galaxy project server. The location of the V3C-T5-seq relative to that of published fragile site locations was computed by calculating the relative location of peaks in 2 Mb windows divided into 250 kb windows. V3C-T5-seq peaks containing more than 1,000 reads in two independent biological replicates were selected for *in silico* screening of transcription factor-binding sites. BEDtools was used to obtain the DNA sequence of the intervals, which was used as the input for motif search using MEME. The transcription factor that associated with the 10 most common motifs was searched using FIMO and TOMTOM (68). Statistical significance of the V3C-seq with V3C-T5-seq analysis was performed using Jaccard analysis on BEDtools. The “observed” values were calculated using the intersection of all HBV sites with that of the cccDNA localization sites. This was compared with “permuted” values, where the HBV localization sites were intersected with a randomly generated set of genomic localization sites of the same size and number as cccDNA-associated regions.

### cinqPCR analysis of HBV cccDNA copies

cccDNA molecules were measured using cinqPCR, as described previously (59). For DNA extraction, HepG2-NTCP cells were seeded in 6-well plates at a density of 300,000 cells per well and incubated for the indicated number of days. Cells were lysed in 1.5 mL TE buffer supplemented with 0.1 mL of 10% SDS for 30 minutes at room temperature. To precipitate nucleic acids, 67 µL of 5 M NaCl was added to each sample, followed by incubation at 4°C overnight. Samples were centrifuged at 14,500 × *g* for 30 minutes at 4°C. The resulting supernatant was extracted with an equal volume of phenol:chloroform:isoamyl alcohol (25:24:1). The aqueous phase was collected and mixed with two volumes of 95% ethanol and then incubated at room temperature overnight to allow nucleic acid precipitation. Samples were centrifuged at 3,500 rpm for 15 minutes, washed once with 75% ethanol, and air-dried overnight. Pellets were resuspended in 50 µL TE buffer.

For restriction digestion of DNA extracted sample, 2 μg of extracted DNA was added to a 20 µL restriction digestion mixture containing 7.5 U RecJf, 10 U HhaI, 200 ng pUC18, and 1× CutSmart buffer. Samples were incubated at 37°C for 15 minutes and then at 42°C for 15 minutes; this cycle was repeated four times. Reactions were heat-inactivated at 80°C for 20 minutes. A 10 µL recircularization mixture comprising 1 µL T4 DNA ligase, 3 mM ATP, and 1× CutSmart buffer was prepared separately and added to the 20 µL digestion reaction. The combined 30 µL reaction was incubated at 16°C for 2 hours and subsequently heat-inactivated at 80°C for 20 minutes. For linearization, 10 U of XbaI in 1× CutSmart buffer to a combined volume of 5 µL was added to the 30 µL sample and incubated at 37°C for 1 hour. The reaction was heat-inactivated at 80°C for 20 minutes and stored at 4°C until further use.

### EdU labeling coupled with immunofluorescence imaging

HepG2-NTCP and HepaRG cells were plated on coverslips in 6-well plates and allowed to adhere overnight before being infected with HBV at the indicated MOIs for 5 days. EdU labeling was carried out using 10 mM EdU stock solution diluted to a final concentration of 20 µM for 2 hours. Cells were fixed using 4% PFA for 15 minutes at room temperature, washed with PBS, and permeabilized with 0.5% Triton X-100 for 20 minutes at room temperature. A volume of 500 mL of the Click-it Reaction Cocktail (containing 1× Click-iT EdU reaction buffer, copper sulfate, Alexa-Fluor-488 azide, and EdU buffer additive) was added to each sample and incubated for 30 minutes and then washed with PBS. Samples were washed in PBS mounted onto coverslips using DAPI-containing Fluoromount. Samples were imaged on a Leica confocal microscope with 63× oil immersion objective.

## Data Availability

All high-throughput sequencing data have been deposited in the Gene Expression Omnibus (GEO) repository and are publicly available under the accession number GSE261927.

## SUPPLEMENTAL MATERIAL

Figure S1 jvi.01014-25-s0001.tiff Quantification of cccDNA over time course.

Figure S2 jvi.01014-25-s0002.tiff HBV genome with the BglII and NlaIII sites indicated.

Figure S3 jvi.01014-25-s0003.pdf HBV localization to host cellular sites genome-wide.

ASM does not own the copyrights to Supplemental Material that may be linked to, or accessed through, an article. The authors have granted ASM a non-exclusive, world-wide license to publish the Supplemental Material files. Please contact the corresponding author directly for reuse.
