# Supplementary figures and images for: Association of hepatitis B virus genomes with active chromatin hubs challenges host replication fidelity, leading to DNA damage
Source: J Virol. 2025 Oct 10;99(11):e01014-25. doi: 10.1128/jvi.01014-25 (PMC12646004; doi:10.1128/jvi.01014-25)

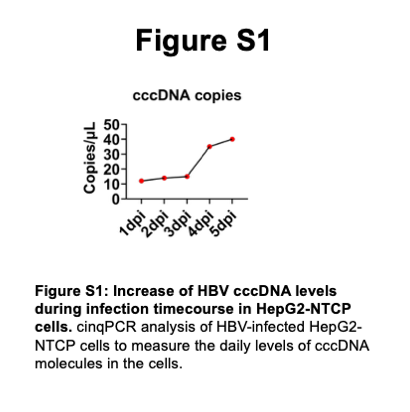

Supplement: Figure S1 — Quantification of cccDNA over time course. [file jvi.01014-25-s0001.tiff]

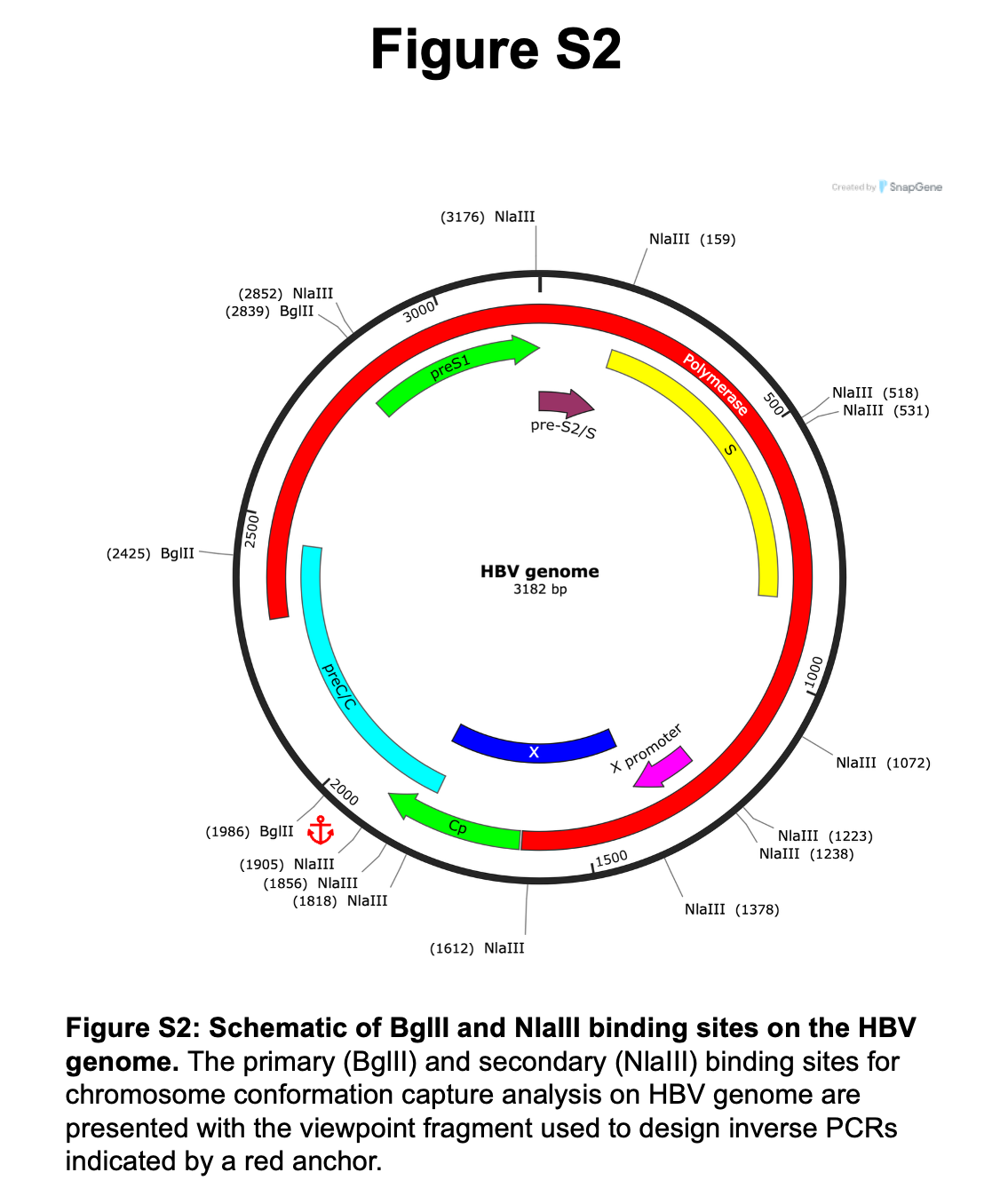

Supplement: Figure S2 — HBV genome with the BglII and NlaIII sites indicated. [file jvi.01014-25-s0002.tiff]
