# Supplementary material for: Association of hepatitis B virus genomes with active chromatin hubs challenges host replication fidelity, leading to DNA damage
Source: J Virol. 2025 Oct 10;99(11):e01014-25. doi: 10.1128/jvi.01014-25 (PMC12646004; doi:10.1128/jvi.01014-25)
Supplement: Figure S3 — HBV localization to host cellular sites genome-wide. [file jvi.01014-25-s0003.pdf]

Figure S3

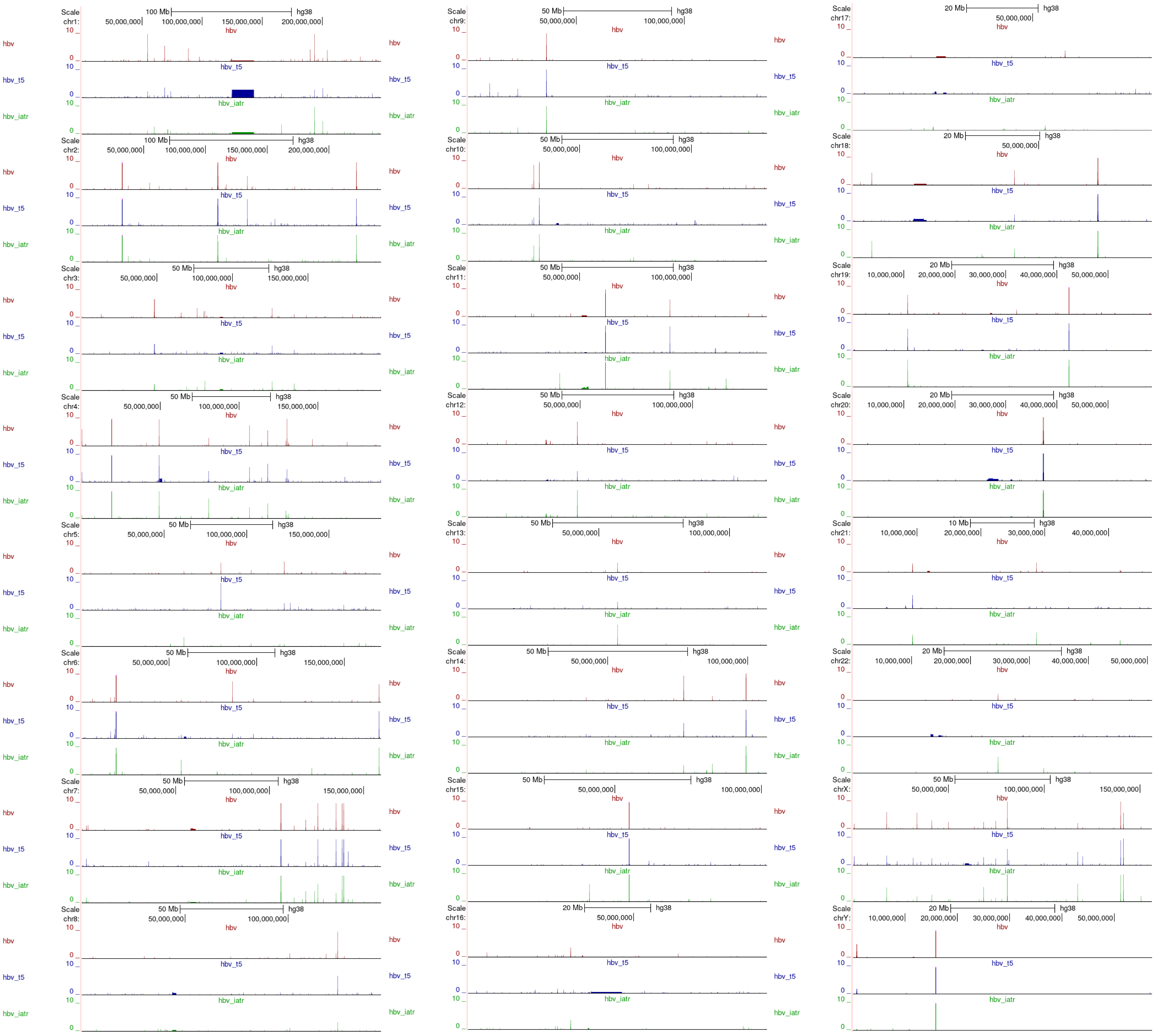

Figure S3: HBV localization to host cellular sites genome-wide. Genome-wide occupancy of HBV localization monitored by V3C-seq (top), localization in the presence of ATR inhibitor (bottom) and V3C-T5-seq peaks (middle) in Hep-G2-NTCP cells infected with HBV at 5 dpi with the viewpoint on the HBV's BglII restriction enzyme site.
